# Annual Report of the Council

**Published:** 1858-07

**Authors:** 


### ANNUAL REPORT OF THE COUNCIL.

[*Presented on Monday, April 5th*, 1858.]

The Council have the honour to submit the following Report to the Members of the Society:—

### MEMBERS OF THE SOCIETY.

During the last twelve months, three Resident Members, three non-Resident Members, and two Corresponding Members have been added to the Society; and three Resident Members have withdrawn from the Society; one non-Resident Member and one Vice-President have died.

The following are the present numbers of the various classes of members:—

| | |
|---|---|
| Resident . . . . | 75 |
| Non-Resident . . . | 46 |
| Corresponding . . . | 90 |
| Honorary . . . . | 6 |
| Total . . | 217 |

### PAPERS READ AT ORDINARY MEETINGS.

Those of the members who have attended the ordinary meetings will have observed, that the papers presented during the past year have been interesting and valuable; and that two of them have been the result of closely-watched epidemics—the one occurring in Turkey, and the other in the Cape de Verd Islands.

*Monday, April 6th.*, 1857.—Two papers; one "On the Outbreak of Fever on board the Ottoman ship of the line, *Tesherefieh*," by J. N. Radcliffe, Esq., late of the Ottoman Medical Staff;—and the second, "On Scurvy on board the Ottoman brig of war, *Fezri-Sefet*," by the same author, were read by Dr. McWilliam.

*Monday, May 4th.*—The following papers were read by Dr. McWilliam:—

1. "On the Cholera Epidemy at Fogo, in the Cape de Verd Islands, in 1855." By José Fernandez da Silva Leão, Surgeon in the Portuguese Navy, and Colonial Surgeon in the Cape de Verd Archipelago.

2. Extract from the Report of Dr. Agostinho Ramos de Carvalho, Chief of the Medical Staff, and President of the

Board of Heath, on the subject of the Cholera Epidemy at Fogo.

3. "Observations on Cholera, as it appeared in Rio de Janeiro in 1855-6." By George Lee, M.D., of Rio.

4. "On Cholera at Para, in the Brazils, in 1855-6." By Samuel Vines, Esq., Her Majesty's Consul, Para.

*Monday, June 1st.*—A paper "On the Mortality from Eruptive Fevers at various Periods of Life," by J. W. Tripe, M.D.; was read by the author.

*Monday, July 6th.*—A paper "On the History of Gaol Fever in England," by F. C. Webb, M.D.; was read by the author

*Monday, August 3rd.*—A paper "On the Causes influencing the Diffusion and Type of Epidemics," by W. I. Cox, Esq., M.R.C S., and Corresponding Member of the Epidemiological Society; was read by Dr. McWilliam.

*Monday, November 2nd.*—A paper "On the Study of Epidemic Diseases, illustrated by the Pestilences of London," by E. H. Greenhow, M.D.; was read by the author.

*Monday, Dec. 7th.*—A paper "On the District Mortality of London," by W. Odling, M.D., was read by the author.

*Monday, Jan. 4th,* 1858.—A paper "On the Causes which influence the Arrest or Spread of Cholera," by F. H. Johnston, Esq., M.R.C.S., of Bishop's Wearmouth; was read by Dr. McWilliam.

*Monday, Feb. 1st.*—A paper "On the Influence of Drainage and Water Supply on Public Health," by John Snow, M.D.; was read by the author.

*Monday, March 1st.*—A paper "On the Investigation of Epidemic Diseases by Experiment," by B. W. Richardson, M.D.; was read by the author.

### INVESTIGATION OF EPIDEMIC DISEASES.

The progress of epidemic disorders in various parts of the globe has, during the last twelve months, been such as to suggest the necessity of more closely investigating this class of diseases.

The President, in his introductory address at the opening of the session, alluded, as you will recollect, to the appearance of cholera at Hamburgh, and at several of the Baltic ports; and to the occurrence of some cases of the same disorder at Stratford and Poplar.

*Yellow Fever* has, in the course of the year, again broken out in various places in the West Indies. It continues persistently at St. Thomas, keeping at times comparatively quiet, until it finds food for a fresh irruption in the new comers arriving in the Mail Company's steam-vessels, in several of which it has proved very fatal.

Newcastle, in Jamaica, a military station, which, from its height above the sea-level, had until of late years been considered beyond the reach of the scourge, has again been visited by this fever.

Rio de Janeiro, invaded for the first time for at least one hundred and twenty years by yellow fever in 1849, was, by the last accounts, being again devastated by this pestilence. A fever of a most fatal character (causing a mortality of 5000 in 13,000 attacked), most probably yellow fever, prevailed in Lisbon from some time in last autumn, until within the last two months. The nature and history of this fever has been investigated by M. Guyon and Dr. Lyons; but the Council are not aware that the report of either of these gentlemen has as yet been made public.

A short communication on the subject of yellow fever at Newcastle, Jamaica, has been received from Dr. Lawson, Deputy Inspector of Hospitals, and principal medical officer on the station.

### HEALTH OF THE ARMY IN INDIA.

Amidst the disastrous events which have of late rivetted the world's attention to India, and given rise to the necessity of accumulating in that country military masses of unacclimated Europeans, it is, at least, matter of congratulation, if not of triumph, for the progressive appreciation of sanitary rules by military commanders, that fearfully destructive epidemic disease has not been allowed, either through neglect or ignorance, to add to those evils which are inseparable from war. Though these disasters have necessarily interrupted the Society's intercourse with its Indian contributors to the subject of epidemic diseases, it is, nevertheless, incumbent on us to mention in this report, that amidst all the marching, hardships, and exposure to which our troops have been subjected, no great outbreak of epidemic cholera has as yet appeared, even among the regiments recently arrived from Europe. How different were the results among our soldiers and seamen sent to the West Indies at the commencement of the last war, when, after predisposition to disease in ill-ventilated transports, and the influence of improper diet, drink, and the want of personal cleanliness, both were allowed to perish from fevers and dysenteries of the most deadly type, brought into destructive action by ill-selected sites of encampment, unsuitable clothing, with bad barrack and hospital accommodation.

It has been seen how much can be done by judicious dietetic regimen, exercise, attention to clothing, careful selection of site, and good barrack and hospital accommodation, to mitigate the diseases incident to Europeans in warm climates, and lessen the liability to epidemic outbreaks.

### LABOURS OF COMMITTEES.

*Sub-Committee on Medical Nomenclature.*—With the view of establishing uniformity in the mode of recording diseases, and thus facilitating statistical and other inquiries, a Sub-Committee of the Hospitals' Committee was appointed in the early part of the session, to frame a new nomenclature of diseases, for adoption by the profession in the public services, as well as in civil life. It was, however, soon evident, that a work of such importance could only be satisfactorily accomplished by the united labours of men of experience and learning from all the corporate medical bodies in the metropolis, and from the various public services. The President of the Royal College of Physicians at once acceded to the proposition of the Sub-Committee, to form a sub-committee of the college, on the widest possible basis; and, accordingly, that sub-committee has been sitting once during each fortnight since the middle of January last. It consists of the President of the Royal College of Physicians, as President; Dr. Druitt, from the Apothecaries' Society; the President of the Royal College of Surgeons, various other Fellows of the Colleges, including Dr. Babington, the President of this Society, and Dr. Sibson, Secretary of the Sub-Committee; Dr. Farr, of the General Register Office; Sir John Liddell, Director-General of the Medical Department of the Navy; Dr. Logan, from the Army and Ordnance Medical Department; Mr. J. R. Martin, representing the East India Company's Service; the Secretary of the Epidemiological Society, etc. etc.

*The Smallpox and Vaccination Committee* have not been idle. The delay which has occurred in passing a measure for the more efficient regulation of public vaccination has given them much anxiety, and they have felt that exertion was called for. It will be in the recollection of the Society, that, rather more than two years ago, the Government introduced into the House of Commons a bill for this purpose, which acknowledged the principles, and carried out many of the suggestions urged by this Committee; and that this bill, though met with objections which the Council (in common, as they believe, with all who have paid attention to the subject) consider futile, and threatened with opposition; yet so far received the favourable consideration of the House, that it passed a second reading without division. From that point, however, to the present time, owing to political events, the alleged pressure of public business, and other causes, no progress whatever has been made; though repeated assurances have been given in answer to representations made by this Council, and to inquiries in Parliament, of the intention of the Government to persevere; and though, in the meanwhile, an independent inquiry, instituted by desire of the Government, by their own medical officer, has confirmed, in every respect, the views advanced by the Society, and has, in the most conclusive way, disposed of the grounds of objection raised.

It is scarcely possible to overstate the evil which has resulted from this delay. Within these two years, thousands upon thousands have suffered from the smallpox, and many thousand persons (chiefly young children) have died therefrom, a large number of whom might, beyond all question, if this bill had been passed when it was first introduced, have been saved. This sickness and mortality are going on from day to day—and there is every reason to expect that they will continue at an increased ratio, for, under the influence of an unsettled state of things, the public vaccinations for the last three years have been declining, while smallpox, as noticed in the last Quarterly Report of the Registrar-General, is becoming more extensively epidemic over the kingdom. Under such circumstances, and to prevent the loss of another session, it seemed highly essential to the Committee, and their view was at once adopted by the Council, that these facts should be strongly represented to the Government and to the House of Commons; and for this purpose, soon after the assembling of Parliament, the President of the Society, accompanied by a deputation, waited on the then Prime Minister, and on the President of the General Board of Health, and a petition to the House of Commons was drawn up, of which Sir John Pakington has kindly taken charge. The change of administration, which took place almost immediately after the interview alluded to, rendered it necessary that the reasons for immediate legislation, submitted to Lord Palmerston, should be laid before Her Majesty's present advisers. This will at once be done—and no further effort will be wanting to secure their attention to this important subject, and the enactment of a bill during the present session. It seems favourable to the hopes which the Council entertain in this respect, that the present Cabinet includes many members to whom the subject is by no means new—the author of the first vaccination law (the Earl of Ellenborough), Sir John Pakington, who had charge of the Vaccination Extension Act in the House of Commons, and who has always taken much interest in the subject, and has undertaken to present the Society's petition; and Lord Stanley, who presided over the section of Public Health at the Social Congress at Birmingham.

There are two observations on the proposed amendment of the Vaccination Laws, which, in the present state of the question, the Council deem it important to make. The first is, that the proposal to provide skilled (which, in this instance, means, of course, medical) supervision of public vaccination has no necessary connexion with the question of compulsory vaccination; and that such supervision is needed, whether there be or be not a compulsory law. Secondly, that the securing such a supervision is all that was implied in the proposal of the Society to make public vaccination a branch of the Board of Health; this branch being simply considered by them to represent the department of the Government having charge of questions affecting the health of the people and the prevention of diseases. Whether this board be maintained, or whether its functions be transferred to a committee of council, or to a department of the Home Office, is quite immaterial to the argument of the Society. It being assumed that there must be somewhere a medical department of Government, it seemed a far simpler course to the Society to place public vaccination under it, than to propose the institution of a separate vaccination department. But this, the Council would observe, is entirely a question for the Government, the demand of the Society being only the provision of efficient medical supervision—a demand, the reasonableness of which, and the necessity of which, have never, so far as they are aware, been called in question.

The reasons for desiring to see an efficient vaccination law in operation in England and Wales are not limited to this division of the empire. The state of Scotland and Ireland is such as earnestly to demand the attention of the Government; and the enactment of an efficient system here will be the first step toward securing a similar advantage for these kingdoms. The state of Scotland is constantly remarked upon by the Registrar-General of that country; and that distinguished medical philosopher, Dr. Alison, justly remarks, that it is a grievance greater than many which disturb the consciences of many of our legislators to allow such a pestilence as smallpox is in Scotland to go on unchecked by the law. In the same way, the Irish Poor Law Commissioners, who have the charge of public vaccination, remark in their last Report on the necessity of providing by legislation for the more effectual carrying out of public vaccination in Ireland; and one of them, writing to the President of the Society, states that the manner in which public vaccination is now carried out in Ireland occasions him continual uneasiness,—and that the next Report will show a falling off in the number of Dispensary vaccinations for the year ending September 30, 1857, compared with the preceding year, of nearly 40,000.

That such neglect should exist is alike disgraceful and appalling. The Epidemiological Society will never cease to agitate this question till they see an adequate remedy applied; if only to aid in the accomplishment of such a purpose as this, they may well rejoice that they have been called into existence.

### THE NATIONAL ASSOCIATION FOR THE ADVANCEMENT OF SOCIAL SCIENCE.

As an evidence of the recognized importance of sanitary science, and of the increased attention that is now being devoted to its study, the Council refer with much satisfaction to the National Association for the Promotion of Social Science; which, as the Society is aware, was instituted in October last, under the presidency of that great champion of social progress, Lord Brougham.

Among the papers of the various sections recently published in the transactions of the Association, none are more interesting or more valuable, than those of the department of Public Health.

In these most instructive documents, we find the following subjects clearly and ably investigated:

A. *Condition of the Public Health.*—"The Rapid Increase of Town Populations," by Dr. Beddoe.

"The Mortality of Birmingham," by Mr. Green.

"The Necessity for a more Analytical Study of Public Health Statistics," by Dr. Greenhow.

"Huddersfield, and its Sanitary Statistics," by Mr. Knaggs.

"Statistics of Health of the Waterguard and Waterside Officers of the Customs," by Dr. M'William.

"Sanitary Measures in a Provincial Town, and their Results," by Mr. May.

"The Influence of Habitation on the Community," by Mr. Michael.

"The Sanitary Condition of the Colliery Population, in the County of Durham," by Mr. Oliver.

"The Composition of Town Populations," by Mr. Welton.

B. *Causes which Modify the Public Health.*—"The River Severn, in Relation to the Pollution of its Stream by Drainage," by Sir Charles Hastings.

C. *Improvement of the Public Health.*—"The Drainage of Towns," by Mr. Austin.

"The Sewerage of Towns," by Mr. Rawlinson.

"The Sewers of Birmingham," by Mr. Pigott Smith.

"Arrangement, Drainage, and Ventilation of Houses, for Working Men," by the Rev. C. H. Hartshorne.

"Parks and Public Places of Recreation for the People," by Mr. Langford.

"Hygiene, as a Branch of Military Education," by Sir John Richardson.

"On an Improved Air-tight Coffin," by Mr. Salt.

"The Disinfection of Sewers," by Dr. Sanderson.

"Public Vaccination in England and Wales," by Dr. Seaton.

"Central and Local Action, in Relation to Town Improvement," by Mr. Tom Taylor.

"The Ventilation of Dwelling Houses, by Means of the Kitchen Fire," by Dr. Wyld.

There are also the following papers on miscellaneous subjects,—viz :

"The Adulteration of Food and Drugs," by Mr. Postgate.

"The Necessity of Sanitary Arrangements for Armies in Barracks, as also in the Field," by Mr Rawlinson.

"The Evidence of the Prolongation of Life, during the Eighteenth Century," by Dr. S. Smith.

The Public Health Department was presided over by the most promising statesman of the age, Lord Stanley, who concluded an address full of practical wisdom, in the following most appropriate language:

"Dry and unattractive as sanitary studies may appear, they belong to the patriot no less than to the philanthropist —they touch very nearly the future prosperity and the national greatness of England. Don't fancy that the mischief done by disease spreading through the community, is to be measured by the number of deaths which ensue. That is the least part of the result. As in a battle, the killed bear but a small proportion to the wounded. It is not merely by the crowded hospitals, the frequent funerals, the destitution of families, or the increased pressure of public burdens, that you may test the suffering of a nation over which sadness has passed. The real and lasting injury lies in the deterioration of the race, in the seeds of disease transmitted to future generations, in the degeneracy and decay which are never detected till the evil is irreparable, and of which even then the cause remains often undiscovered. It concerns us, if the work of England be that of colonisation and of dominion abroad,—if wild hordes and savage races are to be brought by our agency under the influence of civilized man, —if we are to maintain peace, to extend commerce, to hold our own among many rivals alike by arts and arms—it concerns us, I say, that strong hands should be forthcoming, to wield either sword or spade, that vigorous constitutions be not wanting to endure the vicissitudes of climate, and the labours of a settler in a new country. I believe that, whatever exceptions may be found in individual instances, when you come to deal with men in the mass, physical and moral decay necessarily go together, and it would be small satisfaction to know that we had, through a series of ages, successfully resisted every external enemy, if we learnt too late, that that vigour and energy, for which ours stands confessedly preeminent among the races of the world, were being undermined by a secret but irresistible agency, the offspring of our own neglect, against which science and humanity had warned us in vain."

### THANKS OF COUNCIL.

The Council have to acknowledge their obligations to the donors of various presents to the library of the Society; and to the general and professional press for the insertion of abstracts of papers read at the meetings, and for otherwise giving publicity to the proceedings of the Society.

To Dr. Richardson, the editor of the *Sanitary Review*, the thanks of the Society are specially due, for the publication, in full, of the Transactions of the Society in that journal, which, being printed separate from the other parts of the journal, can be bound up in volumes by themselves.

Finally, the Council are desirous of recording their sense of the kindness of the Council of the Royal Medical Benevolent College for the liberal conditions under which the use of their rooms is granted to the Society.

